# Strongly correlated Fermions strongly coupled to light

**DOI:** 10.1038/s41467-020-16767-8

**Published:** 2020-06-12

**Authors:** Kevin Roux, Hideki Konishi, Victor Helson, Jean-Philippe Brantut

**Affiliations:** Institute of Physics, EPFL, 1015 Lausanne, Switzerland

**Keywords:** Atomic and molecular interactions with photons, Ultracold gases, Quantum optics, Quantum simulation

## Abstract

Strong quantum correlations in matter are responsible for some of the most extraordinary properties of materials, from magnetism to high-temperature superconductivity, but their integration in quantum devices requires a strong, coherent coupling with photons, which still represents a formidable technical challenge in solid state systems. In cavity quantum electrodynamics, quantum gases such as Bose-Einstein condensates or lattice gases have been strongly coupled with light. However, neither Fermionic quantum matter, comparable to electrons in solids, nor atomic systems with controlled interactions, have thus far been strongly coupled with photons. Here we report on the strong coupling of a quantum-degenerate unitary Fermi gas with light in a high finesse cavity. We map out the spectrum of the coupled system and observe well resolved dressed states, resulting from the strong coupling of cavity photons with each spin component of the gas. We investigate spin-balanced and spin-polarized gases and find quantitative agreement with ab initio calculation describing light-matter interaction. Our system offers complete and simultaneous control of atom-atom and atom-photon interactions in the quantum degenerate regime, opening a wide range of perspectives for quantum simulation.

## Abstract (web summary)

Atom-photon interaction and their coupling are important to understand correlated and quantum matter. Here the authors show strong coupling between degenerate interacting Fermi gas of ^6^Li atoms and photons.

## Introduction

Strong and coherent light-matter interactions are at the core of emerging quantum technologies, enabling the observation and control of matter at the level of single quanta^1^. In many-body systems, it is reached quantitatively when the collective cooperativity *C*_*N*_ = 4*Ng*^2^/*κ*Γ exceeds unity, i.e. when the fraction of photons coherently scattered into one particular mode of the electromagnetic field, singled-out by a high-finesse resonator, dominates over incoherent loss processes^2^. Here *g* is the coupling strength between a single photon and a single matter excitation, *N* is the number of identical emitters, and *κ* and Γ are the incoherent decay rates of photons and matter excitations, respectively. Strong coupling to light would be highly beneficial for the quantum simulation of interacting Fermions, which display extraordinary properties^3,4^ but where first-principles theoretical calculations are inherently difficult^5^. Indeed, recent theoretical work in both cold atoms and solid state systems suggests that strong coupling would make the realization and control of new quantum states of matter^6–14^ possible, as well as high-precision, quantum-limited measurements^15^.

The strong coupling regime has been achieved with optical photons in various systems with weakly interacting emitters, from semiconductors and 2D material microcavities^16,17^, to atoms and trapped ions^18–20^, including recently thermal Fermionic atoms^21^. Combining evaporative cooling with high-finesse cavities^22–25^ enabled the production of weakly interacting Bose–Einstein condensates strongly coupled with photons. Recently, Bosonic Mott insulators have been dispersively coupled to light^26,27^, representing the only example combining strongly correlated quantum matter and strong light-matter interactions to date.

Here, we report on a unitary Fermi gas in a high-finesse cavity, demonstrating simultaneously the strongest atom–atom interactions and strong light-matter coupling. A Fermi gas of ^6^Li atoms is prepared inside a high-finesse cavity close to a Feshbach resonance, and cooled down to the superfluid regime, as evidenced by phase separation in the presence of a spin imbalance^28–31^. We measure the transmission spectrum of the coupled Fermi gas-cavity system, and observe the emergence of well resolved dressed states and their coherent scaling with atom number, demonstrating strong light-matter coupling. Both features are reproduced by an ab initio calculation accounting for light-matter interactions and several modes of the cavity.

## Results

### Fermi gas preparation

**Fig. 1:**
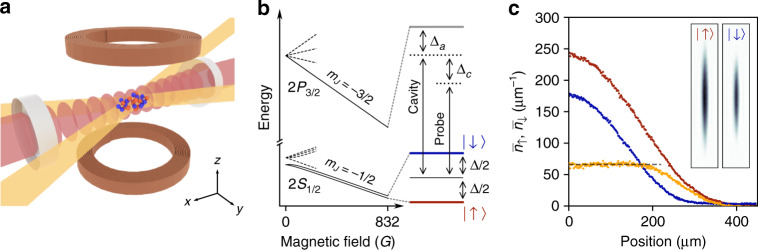
Combining a unitary superfluid with a high-finesse cavity. **a** A two-component Fermi gas is trapped in a crossed-dipole trap aligned onto the mode of a high-finesse optical cavity. A bias magnetic field oriented along the *z*-axis controls the interactions between the atoms. **b** Relevant energy levels of ^6^Li at 832 G including the frequencies of the probe laser and cavity resonance. In the experiment the detunings Δ_*a*_ and Δ_*c*_ are varied independently. **c** Doubly integrated density profiles, ${\overline{n}}_{\uparrow }$ and ${\overline{n}}_{\downarrow }$, along the longitudinal direction after transfer in an elongated trap, for 9.3(5) × 10^4^ and 6.0(3) × 10^4^ atoms in state $\left|\uparrow \right\rangle$ (red) and $\left|\downarrow \right\rangle$ (blue), respectively. The difference between the two doubly integrated profiles (orange) exhibits a plateau, characteristic of a superfluid core surrounded by a polarized shell. The grey dashed line is horizontal to guide the eye. Inset: absorption images (173 × 919 μm) taken along the *z-*axis of both spin states in the elongated trap after 300 μs time-of-flight. The peak line density is 244 μm^−1^.

We produce a paradigmatic example of strongly correlated system, the unitary Fermi gas, inside a high-finesse cavity in the strong coupling regime. The core of the experiment is a 4.13(3)-cm long Fabry-Perot cavity with a finesse of *F* = 4.7(1) × 10^4^, resonant at 671 nm with the dipole-allowed transition frequency of ^6^Li, depicted in Fig. 1a. The experimental sequence starts with a magneto-optical trap, producing laser-cooled ^6^Li atoms within the cavity, followed by all-optical evaporation, first in a cavity-enhanced standing-wave dipole trap^32^, then in a crossed-dipole trap. This yields a two-component Fermi gas in the two lowest hyperfine states denoted $\left|\uparrow \right\rangle$ and $\left|\downarrow \right\rangle$ (Fig. 1b), with tunable populations *N*_*↑*_ and *N*_*↓*_. The whole evaporation takes place under an external magnetic field of 832 G, the location of a broad Feshbach resonance, where atomic collisions are resonant. We obtain a degenerate unitary Fermi gas of typically 2 × 10^5^ atoms in a controlled mixture of the two hyperfine states, held in a trap with an aspect ratio of three, elongated along the cavity direction (see Methods).

One of the hallmarks of strongly interacting Fermi gases is the onset of superfluidity in the deeply degenerate regime^33–35^. We characterize superfluidity by observing phase separation, occurring below the critical temperature in a moderately spin-imbalanced gas^30^. We transfer the atoms into a single-beam trap from the crossed-dipole trap by adiabatically ramping down the power of one arm (see Methods), and measure the doubly integrated density profile difference between the two spin states along the longitudinal direction of the trap^28,29,31^, as shown in Fig. 1c. The difference is constant at the center of the cloud, demonstrating phase separation between a superfluid, fully paired core, and a spin-polarized shell.

### Transmission spectroscopy

We now investigate the coupling of the unitary Fermi gas to the cavity field, characterized by the parameters (*g*, *κ*, Γ) = 2*π* × (0.479, 0.077, 5.872) MHz with *C*_1_ = 2.02, allowing to reach the strong coupling regime for a few atoms. At 832 G, in the Paschen–Back regime, the *σ*^−^ transition $\left|2{S}_{1/2},{m}_{J}=-1/2\right\rangle \, \longrightarrow \, \left|2{P}_{3/2},{m}_{J}=-3/2\right\rangle$ is an ideal two-level system for each spin component. The two spin states are separated by the hyperfine splitting Δ/2*π* = 76.3 MHz, as depicted in Fig. 1b together with the other relevant energy levels. The magnetic field is oriented perpendicular to the cavity axis and we probe this transition with light linearly polarized along the *y*-axis, which reduces *g* by a factor $\sqrt{2}$, down to *g* = 2*π* × 0.339 MHz.

The general spectrum of the coupled light-matter system consists of dressed states, one for each component of the gas and each mode of the cavity. They are coherent superpositions of photonic and atomic states, with a relative weight controlled by the detuning between the atomic and the cavity resonances as well as the light-matter coupling strength. To characterize this spectrum, we inject in the cavity a weak probe beam matched with the TEM_00_ mode and measure its transmission with a single photon counter, with a total detection efficiency of 0.44(5). We perform transmission spectroscopy as a function of the detunings Δ_*c*_ and Δ_*a*_, defined in Fig. 1b. The probe frequency is swept at a rate of 100 MHz/ms and its power is set to keep the intracavity photon number below Γ^2^/8*g*^2^ = 38, the saturation threshold on resonance (see Methods).

**Fig. 2:**
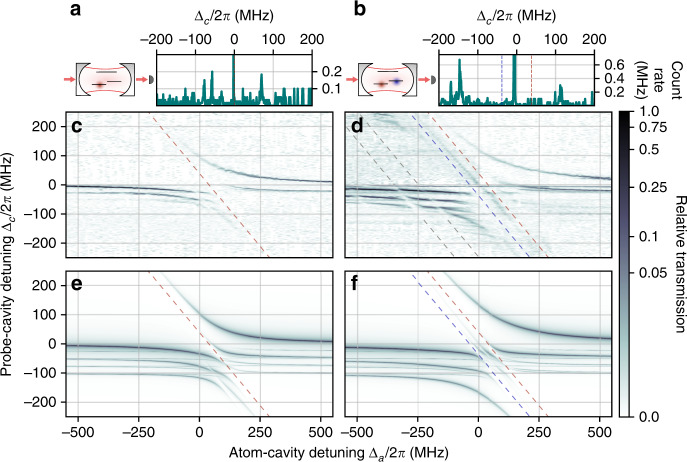
Transmission spectrum of a unitary Fermi gas strongly coupled to light. **a**, **b** Schematic view of the experiment, depicting the three relevant energy levels and cavity transmission signal, averaged over three realizations, as a function of Δ_*c*_/2*π*, for Δ_*a*_/2*π* = 38.2 MHz for a spin-polarized Fermi gas with *N*_*↑*_ = 9.1(7) × 10^4^ and *N*_*↓*_ = 2(1) × 10^3^ (**a**) and for Δ_*a*_ = 0 for a spin-balanced Fermi gas of 2.0(1) × 10^5^ atoms equally populating each spin state (**b**). The values of Δ_*a*_ are centered on the anticrossing feature for both cases. **c**, **d** Coupled-system transmission spectrum as a function of Δ_*c*_/2*π* and Δ_*a*_/2*π* for the spin-polarized (**c**) and spin-balanced Fermi gas (**d**). The transmissions normalized by the maximum observed in each case are displayed in logarithmic scale. The bare atomic transitions for $\left|\uparrow \right\rangle$ and $\left|\downarrow \right\rangle$ are shown with red and blue dashed lines, respectively. The grey dashed lines indicate a weak contribution attributed to molecular states. **e**, **f** Transmission spectra obtained from ab initio calculation accounting for the higher-order transverse mode contributions for the spin-polarized (**e**) and the spin-balanced gases (**f**).

We first measure the cavity transmission in the case of a spin-polarized Fermi gas with *N*_*↑*_ = 9.1(7) × 10^4^ and *N*_*↓*_ = 2(1) × 10^3^. We expect the spectrum to be dominated by the contribution of the state $\left|\uparrow \right\rangle$, demonstrating the simplest case of a single component, non-interacting Fermi gas coupled to light. A typical transmission spectrum for Δ_*a*_ = 0 MHz is shown in Fig. 2a, as a function of Δ_*c*_/2*π*. Figure 2c shows 111 such spectra, in logarithmic scale, for Δ_*a*_/2*π* spanning 1.1 GHz. We observe a prominent anticrossing, characteristic of strong light-matter coupling, as the cavity resonance approaches the atomic one for $\left|\uparrow \right\rangle$ located at Δ_*a*_/2*π* = 38.2 MHz. While the frequencies of the transmission peaks reveal the excitation spectrum, the value of the transmission reflects the weight of the photonic fraction in the respective excitations. For Δ_*c*_ > 0, the dressed state continuously changes from a mostly photonic excitation for large Δ_*a*_, to a mostly atomic excitation as Δ_*a*_ is decreased to negative values.

The spectrum displays a richer structure for Δ_*c*_ < 0, due to the presence of several families of high-order cavity modes, spaced by multiples of 25 MHz on the red side of the bare TEM_00_ mode. Each of these mode families couples to the atomic resonance, producing several transmission peaks appearing in Fig. 2a, associated to dressed states mixing high-order and TEM_00_ cavity modes with the atomic excitations. Even though the coupling efficiency of the probe beam with these high-order modes is less than 10^−3^, the interaction with the finite size atomic cloud allows for scattering of the incident power into these modes, yielding large transmission for dressed states involving them^36,37^. The weaker anticrossing centered at Δ_*a*_/2*π* = −38.2 MHz originates from the coupling of light to the minority population in state $\left|\downarrow \right\rangle$.

Additionally, the probe light also contains a weak linear polarization component along the magnetic field direction, which does not couple to the *σ*^−^ transition and yields a narrow transmission peak between Δ_*c*_/2*π* = 0 and  −10 MHz, as it can be seen in Fig. 2a. This polarization component couples to the *π* transition $\left|2{S}_{1/2},{m}_{J}=-1/2\right\rangle \, \longrightarrow \, \left|2{P}_{3/2},{m}_{J}=-1/2\right\rangle$, located at Δ_*a*_/2*π* = 1.56 GHz.

Similarly, we perform transmission spectroscopy with a spin-balanced gas of 2.0(1) × 10^5^ atoms equally populating both spin states. The results are presented in Fig. 2d, showing two anticrossings as the bare cavity resonance approaches each atomic one, leading to three dressed state branches located above, below and between the two atomic resonances. The low relative transmission of the middle branch results from the large value of the collective Rabi frequency of approximately 2*π* × 150 MHz compared with the hyperfine splitting. Consequently this dressed state is mainly of atomic nature at all detunings, crossing over from state $\left|\downarrow \right\rangle$ to $\left|\uparrow \right\rangle$ as Δ_*a*_ goes from negative to positive.

In contrast with the spin-polarized gas spectrum, the spin-balanced case shows not only strong coupling to the atomic resonances, but also weaker coupling to a set of matter-like excitations on the red side of the $\left|2{S}_{1/2},{m}_{J}=-1/2\right\rangle \, \longrightarrow \, \left|2{P}_{3/2},{m}_{J}=-3/2\right\rangle$ transition, as indicated in Fig. 2d. We attribute these excitations to photo-association into weakly bound states of the 2*S*_1/2_ + 2*P*_3/2_ asymptotic potential. The presence of excited molecular states close to atomic transitions is neither specific to strongly interacting gases nor to a particular spin mixture^38^. However, the ability of photons to couple to these transitions is controlled by the short-range two-body correlations^39,40^. Similar to s-wave scattering, these are suppressed by the Pauli principle in the spin-polarized gas, and are strengthen by pairing and by the Feshbach resonance in the balanced case^41–43^, consistent with our observations.

### Model

We compare our observations with an ab initio calculation accounting for the multimode and multilevel structure of the system. We model atoms in each state as independent two-level systems, disregarding their motional degrees of freedom, distributed in space according to the zero-temperature equation of state, coupled to several families of transverse modes (see Methods). We solve the master equation for the steady state intracavity field including a coherent driving of the TEM_00_ mode, the atomic and cavity decays, without any free parameter. The results are shown in Fig. 2e, f for spin-polarized and spin-balanced gases, respectively. The model well reproduces both the location and shape of the anticrossing of both majority and minority spin states with the TEM_00_. The structure of the dressed states also agree qualitatively for the high-order modes, but the precise location and strength of the various lines strongly depend on the exact position of the cloud, which is only known with limited accuracy. The evolution of the relative transmission along the dressed state branches also agrees qualitatively with the experimental spectrum.

### Scaling with atom number

**Fig. 3:**
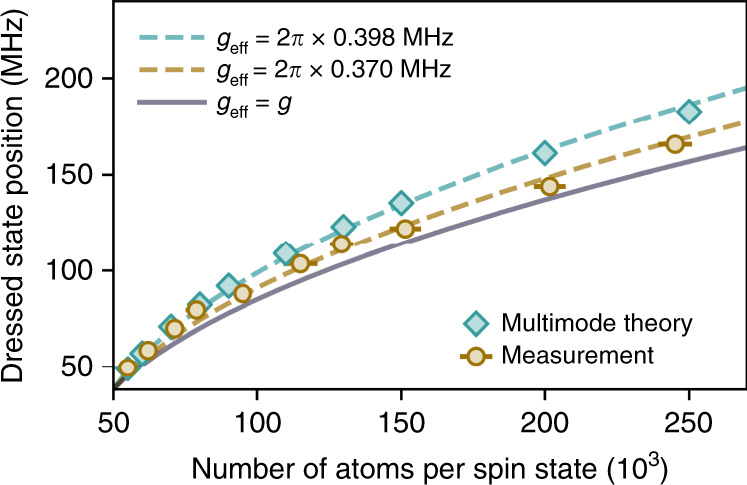
Scaling of the dressed state frequency with atom number. Position of the upper dressed state as a function of atom number in each spin state for a spin-balanced gas, with Δ_*c*_ > 0 and Δ_*a*_ = 0. Orange circles show experimental data and light blue diamonds are obtained from the ab initio theory calculation accounting for all the high-order cavity modes. The lines describe an analytical model with two atomic states and the TEM_00_ cavity mode only. The dashed lines are fits of both experimental data (orange) and theory calculation (light blue) with *g*_eff_ as a free parameter (see text). The purple solid line is the expected scaling for *g*_eff_ = *g* = 2*π* × 0.339 MHz. Error bars are given by the statistical fluctuations over 10 realizations.

We now verify the coherent nature of the light-matter coupling by tracking the position of the upper dressed state for Δ_*a*_ = 0 as a function of atom number per spin state *N* in the spin-balanced case. Atom numbers are measured independently by absorption imaging along the *z*-axis. The results and the predictions of the multimode model are presented together in Fig. 3. The difference between the model and the data is at most 8%. We fit both the experimental data and the theoretical model by $\sqrt{{g}_{{\rm{eff}}}^{2}N+\frac{{\Delta }^{2}}{4}}$, with Δ the hyperfine splitting (lines in Fig. 3), describing two equally populated atomic states coherently and uniformly coupled to a single cavity mode (see Methods). Leaving *g*_eff_ as an adjustable parameter, both the data and theory are well fitted, confirming the coherent nature of light-matter coupling. This yields *g*_eff_ = 2*π* × 0.370 and 2*π* × 0.398 MHz, 8% and 16% larger than *g*, respectively. We attribute these differences to the role of high-order modes, which is slightly overestimated in the ab initio model (see Methods).

## Discussion

A Fermi gas with both strong light-matter and atom–atom interactions opens many unexplored directions. In the dispersive regime, the cavity mediates a long-range interaction^19^, making this system an ideal platform to explore the interplay between Fermi statistics, density wave ordering^44–46^ and superfluidity^47^, a paradigm of competing orders also encountered in strongly correlated materials^48^. The cavity provides a controlled dissipation channel, a promising tool to prepare new correlated states of Fermions^49^. Correspondingly, the photon leakage realizes a weak continuous measurement whose back-action can compete with short-range interactions^50^ and allow for the implementation of feedback protocols^51^. Lastly, our evidence for molecular transitions addressed by cavity photons offers the possibility to control interactions^52,53^ using dynamical, quantized fields, opening a new class of models to quantum simulation.

## Methods

### Preparation of a degenerate unitary Fermi gas in a high-finesse cavity

The experimental sequence starts with ~4 × 10^8 ^^6^Li atoms captured in a magneto-optical trap (MOT). After 3 s loading, the MOT is compressed, optically pumped into the *F* = 1/2 hyperfine manifold, and about 10^7^ laser-cooled atoms are loaded into a cavity-enhanced standing-wave dipole trap at 1064 nm. The cavity has a finesse of  ~3000 at 1064 nm, providing a power build-up of  ~1000. We use the TEM_10_ cavity mode with a vertical nodal line for dipole trapping, in order to mitigate the thermal effects of the 1064 nm light on the cavity stability.

The bias magnetic field is ramped up to 832 G within 250 ms, before the evaporative cooling starts. The first evaporation ramp in the standing-wave trap lasts 300 ms. Approximately 1.5 × 10^6^ atoms are then transferred in the running-wave crossed-dipole trap by turning off the standing-wave dipole trap. The crossed-dipole trap is made of two noninterfering, 1.5 W, 1064 nm laser beams focused to waists of 32(1) μm and intersecting at the cavity mode waist, with an angle of 35^∘^. A 350 ms long linear ramp reduces the power down to 80 mW, which completes the evaporation, then followed by a recompression stopping the evaporation and maximizing the coupling with the 45.0(3) μm waist TEM_00_ cavity mode at 671 nm. For the measurements, the trapping frequencies are (*ω*_*x*_, *ω*_*y*_, *ω*_*z*_)/2*π* = (300(30), 924(40), 902(11)) Hz, yielding a Fermi energy of *E*_F_/*h* = 53(5) kHz for *N*_*↑*_ = *N*_*↓*_ = 1.00(5) × 10^5^, where *h* is Planckʼs constant.

The populations in states $\left|\uparrow \right\rangle$ and $\left|\downarrow \right\rangle$ are tuned during the evaporation process. Balancing the populations between the two spin states is achieved using incomplete Landau–Zener radio-frequency (RF) sweeps during the entire evaporation, and the total atom number can be varied from 1 × 10^3^ to 4 × 10^5^ by adjusting the evaporation endpoint. A controlled spin-imbalance is introduced by removing the RF sweeps and introducing losses in the $\left|\downarrow \right\rangle$ population using the *p*-wave Feshbach resonance located at 214.9 G before evaporation. The largest population difference corresponds to *N*_*↑*_ = 9.1(7) × 10^4^ and *N*_*↓*_ = 2(1) × 10^3^.

The length of the cavity is stabilized during the entire experimental sequence using the Pound–Drever–Hall stabilization technique with an additional laser at 532 nm, for which the cavity has a finesse of approximatively 3000, on the TEM_02_ mode. The power injected in the cavity creates a peak lattice potential of 46 nK = 8 × 10^−3^*E*_*R*_/*k*_*B*_, with *E*_*R*_ the recoil energy and *k*_*B*_ the Boltzmann constant.

### Superfluidity characterization

In our crossed-dipole trap, the large density and small cloud size preclude in-situ imaging, thus preventing a direct measurement of the equation of state. To circumvent this problem, we transfer the Fermi gas from the crossed-dipole trap to a single-beam dipole trap by adiabatically turning off one of the arms after the entire evaporation is completed. The atoms are then held in the elongated trap for another 250 ms to ensure thermal equilibrium. This trap has trapping frequencies 2*π* × (28.5(2), 846(4), 846(4)) Hz, with longitudinal trapping ensured by the magnetic field curvature. We image the atoms in either spin state after a time-of-flight of 300 μs, short compared with the longitudinal trap frequency, which increases the transverse size above the resolution of our imaging system. Integration along the transverse direction yields fig. 1c.

### Multimode theoretical model

The model contains two sets of two-level systems describing the two hyperfine states $\left|\uparrow \right\rangle$ and $\left|\downarrow \right\rangle$, with resonance frequencies (*ω*_*e*,*↑*_, *ω*_*e*,*↓*_)/2*π*. We model the high-order modes of the cavity by four families of TEM_*mn*_ modes, with *n* + *m* ∈ {11, 22, 33, 44}, in addition to the TEM_00_, so that 115 modes are accounted for in total. Successive families are spaced by 25 MHz, and all modes within one family are degenerate. We designate the mode frequencies by *ω*_*ν*_/2*π*, *ν* labeling each individual mode.

Introducing the annihilation operators ${\hat{a}}_{\nu }$ for the cavity modes and the Pauli matrices ${\hat{\sigma }}_{z}^{(i,\lambda )}$ for atom *i* in state *λ* = *↑*, *↓*, the free Hamiltonian reads (with *ℏ* = 1):

1 $${\hat{H}}_{0}=\sum_{\nu }{\omega }_{\nu }{\hat{a}}_{\nu }^{\dagger }{\hat{a}}_{\nu} +\sum_{i,\lambda }{\omega }_{e,\lambda }\frac{1+{\hat{\sigma }}_{z}^{(i,\lambda )}}{2}.$$

We describe the light-matter coupling within the rotating wave approximation as

2 $${\hat{H}}_{{\rm{int}}}=-i\frac{{\Omega }_{0}}{2}\sum _{\nu ,i,\lambda }\left({\hat{a}}_{\nu }{f}_{\nu }({r}_{i}){\hat{\sigma }}_{+}^{(i,\lambda )}-hc\right)$$

where *hc* stands for Hermite conjugate, and we have introduced the Rabi frequency Ω_0_ for the TEM_00_ mode, calculated from spectroscopic data of ^6^Li and the Breit–Rabi formula. *r*_*i*_ is the position of atom *i* considered here as a fixed, classical parameter, and *f*_*ν*_(*r*) are the mode functions, defined as $\sqrt{V}{u}_{\nu }(r)$, *V* is the mode volume common to all modes, and the functions *u*_*ν*_ are an orthonormalized set. We also include the external driving at frequency *ω*/2*π*:

3 $${\hat{H}}_{{\rm{drive}}}=i\sqrt{\kappa }\sum_{\nu }{{\mathcal{F}}}_{\nu }\left({\hat{a}}_{\nu }{e}^{-i\omega t}-{\hat{a}}_{\nu }^{\dagger }{e}^{i\omega t}\right)$$

where the driving strength of mode *ν* is ${{\mathcal{F}}}_{\nu }$.

We then search for the steady state solution of the master equation including cavity decay and spontaneous emission. We neglect quantum fluctuations and atom-field correlations, thus replacing the field operators by coherent amplitudes *α*_*ν*_ and $\langle {\hat{\sigma }}_{z,\pm }^{(i,\lambda )}\rangle$. In the low saturation approximation, we further have $\langle {\hat{\sigma }}_{z}^{(i,\lambda )}\rangle \sim -1$ and $\langle {\hat{\sigma }}_{-}^{(i,\lambda )}\rangle \sim \frac{{\Omega }_{0}/2}{\Gamma /2+i{\Delta }_{\lambda }}$, with Δ_*λ*_ the detuning between the driving and the resonance for state *λ*.

This way, we eliminate the atomic degrees of freedom and obtain a set of algebraic equations for the field amplitude in mode *ν*:

4 $$-\frac{{\Omega }_{0}^{2}}{4}\sum _{\mu }\left(\sum_{\lambda }\frac{{\int}_{V}{d}^{3}r{n}_{\lambda }(r){f}_{\nu }(r){f}_{\mu }(r)}{\frac{\Gamma }{2}+i{\Delta }_{\lambda }}\right){\alpha }_{\mu }-\sqrt{\kappa }{{\mathcal{F}}}_{\nu }=(i{\delta }_{\nu }+\frac{\kappa }{2}){\alpha }_{\nu }$$

where the index *μ* runs over all the modes, and we have replaced summation over the positions of the atoms by an integral over the density distribution *n*_*λ*_(*r*) of atoms in state *λ*. This highlights the essential role of the finite size of the cloud in redistributing the photons between different cavity modes.

The total light intensity in the cavity ∑_*ν*_∣*α*_*ν*_∣^2^ is shown in Fig. 2. In the linear regime where the model is valid this is equivalent to the outgoing photon flux up to a trivial normalization.

The overlap integrals with each of 115 modes are calculated using the equilibrium, zero-temperature equation of state of the unitary and ideal Fermi gases for the balanced and highly polarized cases, respectively. We suppose that the cloud is perfectly centered on the cavity axis, and we use ideal Hermite–Gauss modes.

While the coupling with the TEM_00_ mode is weakly sensitive to small misalignments and mode shape imperfections, the predictions for higher-order modes are much more sensitive. In particular, the detailed redistribution of photons among the modes changes upon varying the position of the cloud by a fraction of the cloud size. We also observed deviations of the high-order mode profiles compared with ideal Hermite–Gauss modes, such as distortion of the nodal lines, which could be due to cavity mirrors misalignments. As a result, the mode volume for higher transverse modes is likely larger than theory would predict, a possible source of the overestimation of their role in the spectrum.

### Analytical model

To provide further physical intuitions, we also compared our data with an analytical model, generalizing the Tavis–Cummings model to a balanced mixture of two independent internal states with resonance frequencies (*ω*_*e*,*↑*_, *ω*_*e*,*↓*_)/2*π*, with total atom number 2*N*_0_. The model’s Hamiltonian reads

5 $$\hat{H}={\omega }_{e,\uparrow }{\hat{J}}_{z,\uparrow }+{\omega }_{e,\downarrow }{\hat{J}}_{z,\downarrow }+{\omega }_{0}{\hat{a}}^{\dagger }\hat{a}+{g}_{{\rm{eff}}}\left({\hat{J}}_{+,\uparrow }\hat{a}+{\hat{J}}_{+,\downarrow }\hat{a}+hc\right)$$

with *ω*_0_ the resonance frequency of the cavity mode and *g*_eff_ the light-matter coupling strength. Here the ${\hat{J}}_{\mu ,\lambda }$, *μ* = *z*, ±  are collective spin operators for atoms in state *λ*^1^. In the low saturation regime, the Holstein–Primakoff transformation allows to rewrite the Hamiltonian in terms of Bosonic operators ${\hat{b}}_{\lambda }$ describing individual, noninteracting collective optical excitations shared among atoms in state *λ*:

6 $$\hat{H}={\omega }_{0}{\hat{a}}^{\dagger }\hat{a}+{\omega }_{e,\uparrow }{\hat{b}}_{\uparrow }^{\dagger }{\hat{b}}_{\uparrow }+{\omega }_{e,\downarrow }{\hat{b}}_{\downarrow }^{\dagger }{\hat{b}}_{\downarrow }+{g}_{{\rm{eff}}}\sqrt{{N}_{0}}\left({\hat{b}}_{\uparrow }^{\dagger }\hat{a}+{\hat{b}}_{\downarrow }^{\dagger }\hat{a}+hc\right)$$

where constant terms have been dropped. For *ω*_0_ − *ω*_*↑*_ = − (*ω*_0_ − *ω*_*↓*_) = Δ/2, the normal modes of this coupled harmonic oscillator model have frequencies ${E}_{0,\pm }=0,\pm \sqrt{2{g}_{{\rm{eff}}}^{2}{N}_{0}+{\Delta }^{2}/4}$.

This supposes that all the atoms are maximally coupled to the field. In practice, due to the cosine longitudinal mode shape, the number of atoms coupled to the field is reduced by a factor of 2, yielding the fit function used in the main text.

### Cavity transmission spectroscopy

The probe laser is locked onto a transfer cavity and narrowed-down to a linewidth ≤10 kHz using the Pound–Drever–Hall stabilization technique. Its absolute frequency is regulated using a wavemeter referenced onto a laser frequency-stabilized by saturated absorption spectroscopy on the $\left|2{S}_{1/2}\right\rangle \, \longrightarrow \, \left|2{P}_{3/2}\right\rangle$ transition of ^6^Li.

For one realization of the experiment, we fix Δ_*a*_ and sweep the probe laser frequency by  ±25 MHz within 500 μs, using a broadband acousto-optic modulator, thereby covering a 50 MHz range in Δ_*c*_/2*π*. A fast sweep rate is necessary in order to minimize the effect of atomic motion during the measurement. Over such a scan the probe power varies by at most 10%. The detected signals are averaged over three realizations. The frequency sweep rate is larger than *κ*^2^/2*π*, so that the field will not reach the steady state in the absence of atoms. However, based on the analytical model we expect the decay rate for the dressed state

7 $$\frac{\kappa +\Gamma {N}_{0}{\Omega }_{0}^{2}/2{\Delta }_{a}^{2}}{1+{N}_{0}{\Omega }_{0}^{2}/2{\Delta }_{a}^{2}}$$

to be dominated by the atomic decay rate for all the parameters covered in the experiment. This ensures that steady state conditions are realized during a measurement. This may, however, not be the case for weakly coupled higher-order modes and for the weak *π* polarization contribution.

We estimate the average intracavity photon number, for the maximal count rate detected with the cavity set resonant with either of the two atomic states. It assumes the steady state, and loss in the mirrors inferred from a comparison between the measured finesse and the independently measured transmission of the mirrors. This leads to a mirror transmission of 40 ppm and loss of 25 ppm yielding an average intracavity photon number of 10. This ensures we work below saturation.

## Data Availability

The datasets generated and/or analysed during the current study are available from the corresponding author on reasonable request.
